# Online exposure to marriage information and marriage expectations of Generation Z in China: The roles of marriage value and relative information exposure

**DOI:** 10.1371/journal.pone.0334596

**Published:** 2025-10-27

**Authors:** Zhikang Wang, Hua Yang, Yujie Jiang

**Affiliations:** 1 School of International and Public Affairs, Shanghai Jiao Tong University, Shanghai, China; 2 School of Marxism, Chongqing University, Chongqing, China; 3 School of Public Policy and Administration, Chongqing University, Chongqing, China; Public Library of Science, UNITED KINGDOM OF GREAT BRITAIN AND NORTHERN IRELAND

## Abstract

**Purpose:**

The marriage expectations of Generation Z youth in China, the main force of marriage currently and over the next decade, are declining. In this study, several moderated mediation models were constructed to explain the effects of online exposure to marriage information on the marriage expectations of Gen Z from an information processing perspective.

**Participants and methods:**

A total of 1,390 questionnaires, based on the Online Exposure to Marriage Information Scale, Relative Information Exposure Scale, Marriage Values Scale, and Marriage Expectation Scale, were collected from Gen Z youth across 31 provincial administrative regions in China. Preliminary data analysis was conducted using SPSS 24.0, and the mediation effect test and moderated mediation effect test were conducted using Process 3.5.

**Results:**

The results indicated that (1) online exposure to marriage utility information was positively related to marriage intention, and online exposure to marriage cost information was negatively related to marriage intention; (2) marriage values mediated the relationship between online exposure to marriage information and marriage intention and that between online exposure to marriage information and expected marriage age; and (3) relative utility and cost information exposure played opposite moderating roles in the relationship between online exposure to marriage information and marriage intention, ultimately inhibiting the marriage intention of Chinese Generation Z.

**Conclusion:**

Different types of online exposure to marriage information and the relative information exposure of Chinese Generation Z individuals have varying impacts on their marriage values and marriage expectations. Policy-makers and online platforms should enhance supervision and guidance on online marriage information. Additionally, Generation Z youth should adjust their relative exposure to cultivate healthy marriage values.

## 1 Introduction

The family is considered the fundamental unit of society, with marriage serving as its cornerstone. However, in recent years, the age of first marriage in China has steadily increased, resulting in a decline in the marriage rate. According to statistics from the Ministry of Civil Affairs of the People’s Republic of China, the number of registered marriages has been decreasing annually since 2013. The figures decreased from 13.47 million pairs in 2013 to 6.833 million pairs in 2022, with the marriage rate reaching a new low of 4.8 per 1,000, the lowest since 1985 [1]. This trend of late marriage is accompanied by late childbearing, which contributes to a decline in China’s total fertility rate from 1.58 in 2017 to 1.07 in 2022. Consequently, the ultralow marriage and fertility rates have become significant challenges for Chinese society. Generation Z (Gen Z), defined as individuals born between 1995 and 2009, is currently at marriageable age, with ages ranging from 15–29 years. This demographic accounts for more than half of the current registered marriages and is expected to be the primary group entering marriage in the next decade. Marriage expectation, as subjective anticipation and predisposed judgment individuals form regarding whether and when to marry, represents the likelihood of an individual’s transition from unmarried to married status [2,3] and is predictive of an individual’s future marital behavior. However, research has indicated that the marriage intentions of China’s Gen Z are on a declining trend, the expected marriage age is increasing, and phenomena such as anxiety about marriage, fear of marriage, and a preference for remaining unmarried are becoming increasingly common [4]. The Chinese government has prioritized addressing challenges in youth marriage and family formation through a suite of policy instruments, including housing market interventions and nuptial subsidies designed to reinforce marriage expectations, complemented by the relaxation of two-child restrictions to stimulate fertility rates. However, empirical evidence suggests that this policy portfolio may yield inconsistent outcomes for nuptial and reproductive motivations. For example, research has indicated that after the implementation of the two-child policy, additional increases in housing prices resulted in more negative impacts on marriage rates [5]. Therefore, it is crucial to investigate the factors influencing the marriage expectations of Chinese Gen Z and to elucidate the underlying mechanisms driving these intentions. This understanding can provide empirical evidence to support efforts aimed at increasing the marriage rate and, consequently, the fertility rate.

Existing scholarship has predominantly examined the determinants of marriage intention and expected marriage age through demographic characteristics [6], personal experiences [7], familial influences [3], and policy frameworks [8], but a notable paucity of empirical investigations into Gen Z have been conducted. Gen Z, who has grown up with the internet, is more adept at using it and does so more frequently than other generations. This generation, born in the internet information era and raised with the internet, requires more focused studies. Studies indicate that the duration and degree of internet use can influence the marriage expectations of Gen Z in China [9,10]. However, the diversity of online information means that the effects of different types of internet content on Gen Z’s marriage expectations cannot be generalized. Consequently, studies focusing solely on a specific type of information or merely on internet usage hours have several limitations [11]. Therefore, it is essential to categorize different types of marriage-related internet information and examine their distinct effects on marriage expectations. Marriage value, which reflects the value judgment of Chinese Gen Z on the usefulness of marriage, may be influenced by exposure to various types of marriage information available online, thereby affecting marriage expectations. Research has shown that social information deepens individuals’ values and subsequently influences their attitudes and behaviors [12]. Additionally, according to cultivation theory, media, including online platforms, can cultivate an individual’s perception of reality and foster similar values by disseminating a large amount of information [13,14]. Marriage expectations can be influenced by value judgments and anticipated outcomes of marital behavior [15]. Therefore, different types of marriage information online may impact the marriage expectations of Chinese Gen Z by shaping their marriage values. Online marriage information often emphasizes individualistic values, such as personal emotional satisfaction and individual marital experiences, which can compete with the family-oriented values conveyed by offline marriage information, such as family continuity and mutual support [9]. Compared with offline marriage information, online content may be exaggerated and biased [16], leading to a disparity between reality and the ideal. Consequently, the same type of marriage information from online and offline sources may influence Gen Z’s marriage values in opposing directions. Therefore, relative exposure to online information versus offline information may affect the extent to which online marriage information influences the marriage expectations of Gen Z in China.

This study focuses on the marriage expectations of Generation Z in China. Through questionnaire surveys and statistical analyses of Generation Z youth across 31 provinces in mainland China, this research attempts to explore the impact of exposure to different categories of online marriage information on their marriage expectations. Furthermore, it investigates the mediating role of corresponding categories of marriage values and the moderating effect of relative information exposure in these categories. This study contributes to expanding the research on marriage expectations among Generation Z in China and provides valuable insights for enhancing their marriage expectations.

## 2 Literature review and hypothesis development

The academic understanding of “marriage expectations” can essentially be divided into two categories. One focuses on an individual’s expectations of their spouse and the marital relationship, which pertains to the outcomes that individuals anticipate receiving from marital relationships during a certain period and believe they are entitled to [17]. The other definition of “marriage expectation” primarily concerns the subjective anticipation and predisposed judgment that individuals form regarding whether and when to marry [2]. In this study, marriage expectations refer to psychological expectations and tendencies regarding marital behavior [2], including the marriage intentions and expected marriage ages of individuals. Strong marriage expectations increase the likelihood of engaging in early marital behavior [15]. Previous research has focused mainly on demographic characteristics such as gender, work industry, place of birth, and education level [6]. Additionally, studies have considered individual factors such as economic status [3], premarital sexual behavior [7,18], cohabitation practices [19], subjective well-being [2], and timing attitude [20]. Family-level factors such as family relationships, family values [3], and parental marriage quality [21] have also been explored. Furthermore, the influence of social policy factors, including housing and family policies [8], on marriage expectations has been investigated. Studies have also investigated the relationship between national contexts and marriage expectations. Research findings indicate no significant differences in marriage intentions among young adults in China, Vietnam, and South Korea; however, Chinese youth demonstrate earlier expected marriage ages than their counterparts in the other two countries do [2].

As the first generation to fully grow up in a digitalized environment, the internet has become one of the significant factors influencing the marriage expectations of Generation Z in contemporary society. The marriage expectations of Generation Z are not only shaped by demographic characteristics and influenced by traditional culture, economic and political factors, and family dynamics but also profoundly affected by the frequency and content of marriage-related information encountered in the online environment. Nevertheless, existing research has yet to adequately consider the dynamic interactive relationship between online information exposure and the marriage expectations of Generation Z. Born in the internet era and having grown up alongside the development of the internet, the study of how online marriage information influences the marriage expectations of China’s Generation Z remains an area that warrants further exploration.

### 2.1 Online exposure to marriage information and marriage expectations

Online exposure to marriage information refers to the type and frequency of marriage-related information that Gen Z obtains via the internet [22]. This study categorizes such exposure into two types: online exposure to marriage utility information (OEMU) and online exposure to marriage cost information (OEMC) [23,24]. OEMU primarily highlights the fulfillment and happiness that marriage can bring to an individual. It includes four subtypes: online exposure to emotional utility information, security utility information, economic utility information, and family continuity utility information. Conversely, OEMC highlights the potential losses and costs associated with marriage. It includes four subtypes: online exposure to psychological cost information, opportunity cost information, economic cost information, and physiological cost information. According to social information processing theory, individuals continuously interpret external information, which influences their attitudes and behaviors [12]. Cultivation theory indicates that media, including online platforms, can increase an individual’s behavioral intention [13]. Therefore, when Gen Z is exposed to marriage utility information, they tend to interpret the emotional, security, economic, and family continuity utility of marriage, potentially strengthening their marriage intentions and accelerating the expected marriage age. Conversely, OEMCs may lead them to interpret the psychological, opportunity, economic, and physiological costs of marriage, potentially weakening their marriage intentions and delaying the expected marriage age.

Based on the above analysis, this study proposes the following hypotheses:

Hypothesis 1a: OEMU positively affects Chinese Gen Z’s marriage intention.
Hypothesis 1b: OEMU negatively affects Chinese Gen Z’s expected marriage age.
Hypothesis 1c: OEMC negatively affects Chinese Gen Z’s marriage intention.
Hypothesis 1d: OEMC positively affects Chinese Gen Z’s expected marriage age.

### 2.2 The mediating role of marriage value

Marriage value constitutes a conceptual framework through which an individual assesses the significance of marital behavior based on their individual needs [25]. This framework encompasses perceptions and attitudes toward marriage, including both the utility and cost values associated with it [26]. In this study, the focus is on Gen Z’s value judgments regarding marriage, which form a multidimensional and multilevel cognitive system. Specifically, marriage utility values include aspects such as emotional, security, economic, and family continuity. Conversely, marriage cost values encompass factors such as psychological, opportunity, economic, and physiological costs.

On the one hand, online exposure to marriage information can significantly influence the marriage values of Gen Z [9]. Values are dynamic and malleable, evolving with different experiences and changing as these experiences change [27]. When Gen Z is exposed to specific types of online marriage information, they interpret and absorb the information, which further reinforces and shapes their marriage values. According to cultivation theory, prolonged exposure to online media results in values that align closely with the messages conveyed by the media [28]. Therefore, higher frequencies of exposure to certain types of online marriage information will have a stronger influence on Gen Z’s marriage values. In addition, in the social context of Generation Z’s upbringing, exposure to various types of online marriage information creates different types of directional pull that affect marriage values in diverse ways. Online exposure to marriage information can positively affect marriage values. Specifically, OEMU positively influences marriage utility values, whereas OEMC positively influences marriage cost values.

On the other hand, the marriage values of Gen Z can influence their marriage expectations [3]. Studies have indicated that an individual’s cognition comprises behavioral attitudes, subjective norms, and perceived-behavioral control [15]. These elements collectively influence individuals’ behavioral willingness and intention, ultimately affecting their behavioral decision-making and behaviors. Behavioral attitudes refer to value judgments and consequence assessments of behavior [15]. Marriage values reflect basic value judgments and attitudinal sets about the act of marriage, which drive an individual’s behavioral attitudes. Therefore, the marriage utility value reflects a positive value judgment of marital behavior, namely, that marriage can produce utility for individuals, thereby strengthening an individual’s marriage intention and accelerating the expected marriage age. Conversely, the marriage cost value reflects a negative value judgment of marital behavior, indicating that marriage incurs costs for individuals, thus weakening an individual’s marriage intention and delaying the expected marriage age.

Finally, studies have indicated that the values held by individuals can be deepened in the process of interacting with social information, thereby reinforcing their attitudes and behaviors [12]. Therefore, Chinese Gen Z’s online exposure to marriage information can shape and strengthen their marriage values, subsequently influencing their marriage expectations. Based on the above analysis, this study proposes the following hypotheses:

Hypothesis 2a: Marriage utility values mediate the relationship between OEMU and marriage intention, and OEMU affects Chinese Gen Z’s marriage expectation through marriage utility values.
Hypothesis 2b: Marriage utility values mediate the relationship between OEMU and expected marriage age, and OEMU affects Chinese Gen Z’s expected marriage age through marriage utility values.
Hypothesis 2c: Marriage cost values mediate the relationship between OEMC and Chinese Gen Z’s marriage intention, and OEMC affects Chinese Gen Z’s marriage intention through marriage cost values.
Hypothesis 2d: Marriage cost values mediate the relationship between OEMC and Chinese Gen Z’s expected marriage age, and OEMC affects Chinese Gen Z’s expected marriage age through marriage cost values.

### 2.3 The moderating role of relative information exposure

Online and offline information sources exhibit distinct orientations: online marriage information places greater emphasis on the utility and costs of marriage for individuals, whereas offline information prioritizes the utility and costs of marriage for families [9,16]. Moreover, online marriage information is more prone to exaggeration. Therefore, this study introduces the concept of relative information exposure (RIE) to further explore the formation mechanisms of marriage expectations. RIE measures the extent to which individuals obtain marriage-related information from the internet rather than through offline communication [22]. Specifically, RIE assesses Gen Z’s exposure to specific channels for acquiring and communicating specific types of marriage information. When RIE is greater than 0, they obtain that type of information more from the internet. Conversely, when RIE is less than 0, they obtain that type of information offline more from real life. Like online exposure to marriage information, RIE includes utility information with four subtypes as well as cost information with four subtypes. The impact of online exposure to marriage information on Gen Z’s marriage values may vary depending on their RIE.

Gen Z, often referred to as the digital natives, has refined the concept of marriage through online socialization, emphasizing personal value. They exhibit lower intentions to marry and tend to marry later than previous generations have or sometimes choose not to marry at all [9]. According to the cognitive dissonance theory, when individuals encounter information that contradicts their existing beliefs or behaviors, they experience cognitive dissonance, leading to anxiety and discomfort; thus, individuals need to adjust their cognitive frameworks to alleviate discomfort [29]. Applying this theory, when Gen Z, who generally have low marriage expectations, encounter information about the cost of marriage, they are likely to accept and trust this information. Conversely, when Gen Z is exposed to information highlighting the utility of marriage, they tend to deny these utilities and adopt a skeptical attitude to avoid cognitive dissonance. This reaction can even lead to avoidance behaviors toward such information [30]. Therefore, the moderating effects of relative cost information exposure (RCIE) and relative utility information exposure (RUIE) may differ.

When the moderating effect of RCIE is examined, it is essential to consider the unique characteristics of Gen Z, who tend to exhibit a nonmarriage-oriented attitude and behavior. This demographic trusts information about marriage cost and often overestimates the cost of marriage while being less informed about the actual expenses. According to the social amplification framework of risk, the interactions of risk and risk events with psychology, society, institutions, and culture may strengthen or weaken the public’s perception of risk and their behaviors [31]. Risk is easily amplified in the context of online social media [32]. The social amplification framework of risk reinforces and spreads the “fear of childbearing” in social media, leading women to overestimate the risk of childbearing, thus creating a spiral of “fear of childbearing” [33]. Marriage costs and marriage cost information are similar to those of risks and risk events and thus can be applied to risk amplification framework theory. Gen Z’s perceived risk of high bride price, domestic violence, loss of growth opportunities, and other costs of marriage disseminated by online social media may be further exaggerated and amplified [16,32]. This overestimation of the probability and factual proportion of marital cost events reduces Gen Z’s marriage expectations. Furthermore, when individuals learn more marriage cost information from internet sources, their OEMC increases, whereas the exchange of information on marriage costs with others offline decreases. According to cultivation theory, this bias in estimating the probability of occurrence and the proportion of this type of information results in a deviation of the expected cost from the actual cost, leading to distorted social expectations [34]. This, in turn, reinforces the negative impact of OEMC on Gen Z’s marriage cost values. Therefore, this study proposes the following hypothesis:

Hypothesis 3a: RCIE positively moderates the negative impact of OEMC on Chinese Gen Z’s marriage cost values. In other words, the negative effect of OEMC on marriage cost values is strengthened when the RCIE is high.

Building on Hypotheses 2c, 2d and 3a, this study further proposes the following hypotheses:

Hypothesis 3b: RCIE positively moderates the mediating role of marriage cost values between OEMC and Chinese Gen Z’s marriage intention. Specifically, when RCIE is high, the mediating effect of marriage cost values on the relationship between OEMC and marriage intention is strengthened.
Hypothesis 3c: RCIE positively moderates the mediating role of marriage cost values between OEMC and Chinese Gen Z’s expected marriage age. Specifically, when RCIE is high, the mediating effect of marriage cost values on the relationship between OEMC and expected marriage age is strengthened.

With respect to the moderating role of RUIE, several key points emerge. First, Gen Z, which often tends toward delayed and nonmarriage-oriented behavior, shows inconsistency in its attitudes and behaviors regarding online marriage utility information. This generation is more skeptical of online marriage utility information than of information obtained from offline sources. Consequently, when RUIE is higher, skepticism toward this information intensifies, thereby diminishing its facilitating effect. Second, RUIE reflects Gen Z’s frequency of acquiring and communicating marital information via the internet. However, this frequent engagement with online channels can lead to information overload. Such overload makes it more challenging for individuals to assimilate and comprehend online marriage utility information [35], thereby weakening the positive impact of OEMU on marriage utility values. Finally, marriage utility information obtained and exchanged offline in real life typically represents the characteristics, structure, and actual utility of the local marital market, whereas OEMU may increase an individual’s expectations. When RUIE is high, it can create a discrepancy between expected and actual marriage utility [36]. This discrepancy further weakens the impact of OEMU on marriage utility values. Based on these insights, the following hypotheses are proposed in this study:

Hypothesis 4a: RUIE negatively moderates the positive impact of OEMU on Chinese Gen Z’s marriage utility values. Specifically, the positive impact of OEMU on Gen Z’s marriage utility values weakens when RUIE is high.

Synthesizing Hypotheses 2a and 2b and Hypothesis 4a, this study further proposes the following hypotheses:

Hypothesis 4b: RUIE negatively moderates the mediating role of marriage utility values between OEMU and Chinese Gen Z’s marriage intention. In other words, the mediating role of OEMU on Chinese Gen Z’s marriage intention through marriage utility values weakens when RUIE is high.
Hypothesis 4c: RUIE negatively moderates the mediating role of marriage utility values between OEMU and Chinese Gen Z’s expected marriage age. In other words, the mediating role of OEMU on Chinese Gen Z’s expected marriage age through marriage utility values weakens when RUIE is high.

**Fig 1:**
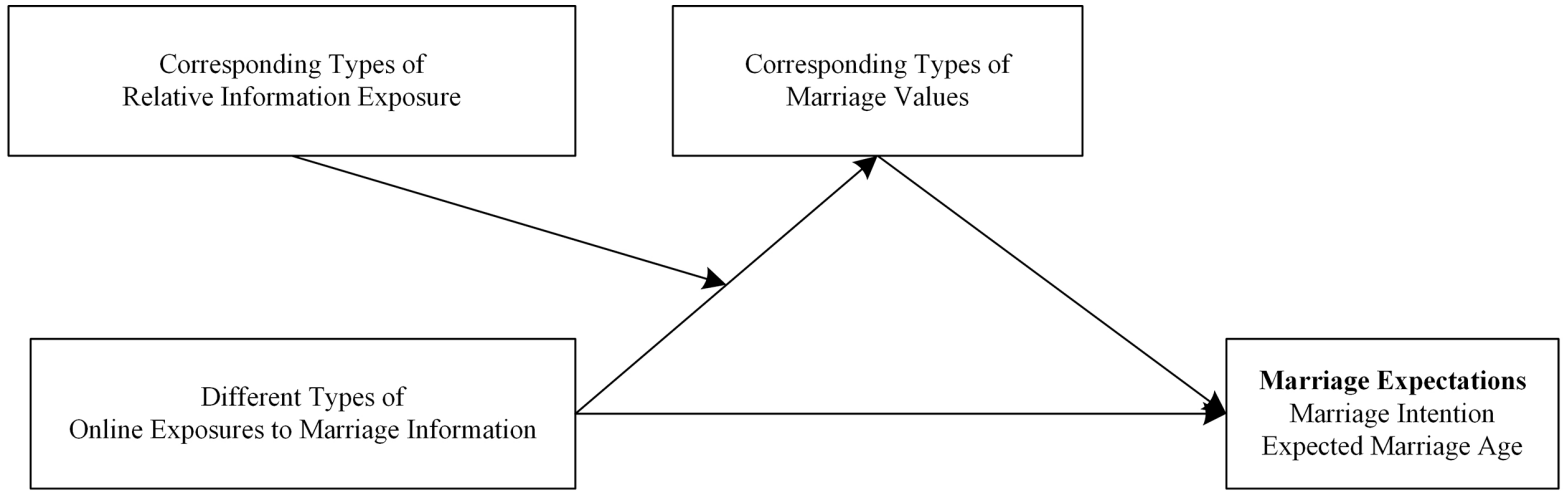
The research model for this study.

The research model of this study is illustrated in Fig 1.

## 3 Methods

### 3.1 Participants and procedure

This study started after approval was obtained from the Institutional Review Board (number CZLS2023062-A). In this study, G Power 3.1 was utilized to conduct a priori power analysis to compute the required sample size. With α = 0.01, power = 0.85, and an effect size of −0.1 [37], the analysis revealed that an effective sample size of 1,296 was needed. Consequently, during the period from November 12, 2023, to January 5, 2024, the study targeted Chinese Gen Z individuals aged between 18 and 30 years. A total of 1,700 questionnaires were disseminated through both offline and online channels. Participants were informed about the purpose, potential benefits, and principles of data privacy and confidentiality of the study, all of which were listed on the first page of the questionnaire. They were also assured that there would be no risks or side effects associated with the study and that they could withdraw from completing the questionnaire at any time without any repercussions. After reading the informed consent form, the participants could voluntarily participate in the survey. When participants explicitly checked the consent box and voluntarily continued to participate, informed consent was considered to be obtained from the participants. For offline distribution, 100 Gen Z individuals from various provinces in China were invited to fill out paper questionnaires at a university, and 98 valid questionnaires were returned; for online distribution, we asked Credamo to send out online invitations to Gen Z individuals. The Credamo platform is a specialized data collection platform in China that offers data services to researchers, similar to Qualtrics and Amazon Mechanical Turk (MTurk). The reason for employing Credamo in our study stems from its stringent data quality assurance protocols and its demonstrated efficacy in precisely targeting Generation Z populations. Therefore, only those who registered on Credamo were invited to complete the online questionnaire, which may have resulted in a lack of representativeness in the research. This study divided the 31 provincial administrative regions in mainland China into northeastern (e.g., Jilin and Liaoning Provinces), eastern (e.g., Shandong and Fujian Provinces), central (e.g., Henan and Anhui Provinces), and western (e.g., Sichuan and Guizhou Provinces) regions. A total of 400 questionnaires were distributed to each region, amounting to 1600 questionnaires. To improve the quality of online data, only real-name authenticated users were allowed to respond. The age limit of the respondents was restricted to between 18 and 30 years, and the location distribution was controlled so that the minimum distance between each respondent was not less than 5 km. Based on criteria such as passing a consistency check, taking more than 120 seconds to complete the questionnaire, and the absence of obvious logical errors in the answers, 1,292 valid online questionnaires were collected. In total, 1,390 valid questionnaires (offline and online) were collected, resulting in a validity rate of 81.76%.

### 3.2 Measures

#### 3.2.1 Marriage value.

Li and Wu [26] rigorously followed standardized scale development procedures and constructed a fertility value scale with excellent reliability and validity. To empirically examine the structural congruence between marriage values and fertility values, this study conducted structured interviews with 97 Chinese Generation Z participants from September to November 2023. The interview protocol included five core questions: “*What factors influence your decision to marry? What contextual elements might accelerate marriage timing? How do you conceptualize the intrinsic value and societal significance of marriage? What perceived costs are associated with marriage commitment? What external pressures could delay marriage?*” Thematic analysis revealed substantial alignment between participants’ responses and the theoretical dimensions outlined in Li and Wu’s fertility values scale [26]. Building upon this empirical validation, we systematically adapted Li and Wu’s fertility value measurement framework to develop a marriage value model and corresponding measurement questionnaire [26]. The marriage values were categorized into marriage utility values and marriage cost values. Marriage utility values were assessed through four dimensions—emotional utility, security utility, economic utility, and family continuity utility—comprising a total of 11 questions. Marriage cost values were evaluated through four dimensions—psychological costs, opportunity costs, economic costs, and physiological costs—with a total of 12 questions. Both scales employed a 5-point Likert scale, where the responses ranged from “strongly agree” (5 points) to “strongly disagree” (1 point). The Cronbach’s alpha coefficients of MUV and MCV were 0.886 and 0.894, respectively. Confirmatory factor analysis indicated that both models demonstrated acceptable fit indices (MUV:χ^2^/df = 5.179, RMR = 0.030, GFI = 0.975, CFI = 0.974, IFI = 0.974, RMSEA = 0.055; MCV: χ^2^/df = 4.914, RMR = 0.056, GFI = 0.972, CFI = 0.975, IFI = 0.975, RMSEA = 0.053).

#### 3.2.2 Online exposure to marriage information and relative information exposure.

In this study, online exposure to marriage information was measured according to the frequency of Gen Z’s exposure to specific types of information via the internet, following Markus Prior’s methodology [38]. The RIE was calculated by subtracting the frequency of offline exchanges pertaining to this information from the frequency of online exposure among Chinese Gen Z study participants [39,40]. Marriage values were categorized into eight types, and online exposure to marriage information reflected how often Gen Z acquired and communicated these eight types of information online. In contrast, offline exposure to marriage information measured the frequency of acquiring the same information through offline interactions. The relative exposure for each type of online information was determined by the difference between the online and offline exposure frequencies. The respondents rated the frequency of their online and offline marriage information exposure on a six-point scale: “never” (1 point), “1–2 times a year” (2 points), “1–2 times in 6 months” (3 points), “1–2 times a month” (4 points), “1–2 times a week” (5 points), and “every day” (6 points). A positive RIE score indicated relative exposure to that type of information from the internet, whereas a negative score indicated relative exposure from offline sources. The reliability of the scales used in this study was confirmed with Cronbach’s alpha coefficients. The coefficients of the OEMU and OEMC scales were 0.843 and 0.831, respectively, and those of the RUIE and RCIE scales were 0.674 and 0.672, respectively.

#### 3.2.3 Marriage expectations.

In accordance with previous studies [2,41], this study measured the marriage expectations of Gen Z by asking respondents “At what age do you plan to marry”. The response options were as follows: “no marriage”, “after 50 years old”, “45 to 49 years old”, “40 to 44 years old”, “35 to 39 years old”, “30 to 34 years old”, “25 to 29 years old”, and “before 24 years old. When participants selected “no marriage”, we coded marriage intention as 0; otherwise, it was coded as 1. We then assigned values from 1–7 to the expected marriage age ranging from “before 24 years old” to “after 50 years old” in sequential order.

### 3.3 Statistical analysis

In this study, SPSS 24.0 and PROCESS macro 3.5 were employed for statistical analysis. The analysis proceeded in several steps. First, Cronbach’s alpha coefficients for the research variables were calculated using SPSS 24.0. Second, descriptive statistics, bias correlation analysis (controlling for control variables), t tests, and a common method bias test were conducted using SPSS 24.0. To investigate differences in the study variables across demographic characteristics, we also employed analysis of variance (ANOVA). Third, we employed regression analysis, logistic regression, and Models 4 and 7 from the PROCESS macro 3.5 developed by Hayes [42] to test the hypotheses. The bootstrap method was set to 5000 iterations with a 95% confidence interval. Demographic variables such as sex, family status, region, family residence, and education level were used as control variables based on the data in recent studies [3,6].

## 4 Results

### 4.1 Common methods bias tests

Given that all the data were self-reported by the study participants and were collected from one source at one time, the potential for common method bias could not be ignored. In this study, we conducted a common method bias test using the Harman one-way test. The results revealed seven common factors with eigenvalues greater than 1. The largest common factor accounted for 22.793%, which was below the critical threshold of 40%, indicating that this study had no significant common method bias.

### 4.2 Descriptive statistics

The descriptive statistics are presented in Table 1. A total of 1,390 valid questionnaires were collected. Among the respondents, 505 (36.3%) were male and 885 (63.7%) were female. The sample distribution was relatively balanced in terms of region. Most participants were from two-parent families (91.2%). Additionally, the general education level was relatively high, with 92.6% holding a bachelor’s degree or above.

**Table 1:** Means of study variables with different demographic characteristics.

		All Samples in This Study (N = 1390)									Subtype with Marriage Intention (N = 1261)		
Item		N	Percentage	OEMU	OEMC	RUIE	RCIE	MUV	MCV	MI	N	Percentage	EMA
Total		1390	100.0%	2.987	3.204	.025	.370	3.667	3.177	91%	1261	100%	1.278
Sex	Female	885	63.7%	2.999	3.369	.055	.493	3.503	3.330	96%	774	61.38%	1.298
	Male	505	36.3%	2.966	2.915	−.028	.155	3.954	2.909	87%	487	38.62%	1.244
Region	Western	329	23.7%	2.925	3.208	.037	.362	3.613	3.258	92%	302	23.95%	1.321
	Central	394	28.3%	3.049	3.253	.073	.420	3.662	3.191	92%	361	28.63%	1.227
	Northeast	291	20.9%	2.857	3.111	.015	.449	3.553	3.264	87%	252	19.98%	1.337
	Eastern	376	27.1%	3.078	3.223	−.029	.265	3.807	3.024	92%	346	27.44%	1.249
Family status	Two-parent	1267	91.2%	2.994	3.206	.022	.362	3.686	3.164	87%	1154	91.51%	1.268
	Others	123	8.8%	2.921	3.187	.055	.455	3.466	3.310	91%	107	8.49%	1.383
Family residence	Urban	937	67.4%	3.028	3.234	.027	.377	3.677	3.138	92%	844	66.93%	1.297
	Rural	453	32.6%	2.903	3.143	.022	.358	3.646	3.257	90%	417	33.07%	1.237
Education level	Masters and PhD	224	16.1%	2.965	3.102	.023	.263	3.649	3.166	89%	208	16.49%	1.361
	Undergraduate	1063	76.5%	3.023	3.259	.043	.413	3.674	3.177	90%	961	76.21%	1.275
	High school or below	103	7.4%	2.670	2.864	−.155	.163	3.630	3.197	93%	92	7.30%	1.120

Notes: MI, marriage intention; EMA, expected marriage age; MUV, Marriage Utility Value; MCV, Marriage Cost Value; OEMU, Online Exposure to Marriage Utility information; OEMC, Online Exposure to Marriage Cost information; RUIE, Relative Utility Information Exposure; RCIE, Relative Cost Information Exposure.

With respect to online exposure to marriage information, the participants had significantly higher OEMC (*M* = 3.204) than the OEMU (*M* = 2.987) (*t* = 7.552, *p* < .001). Male respondents (*p* < .001) and those with a high school education or below (*p* < .01) had a lower OEMC. Conversely, respondents living in towns and cities (*p* < .05) and those with a bachelor’s degree or higher (*p* < .01) preferred browsing OEMU.

With respect to RIE, the RCIE (*M* = .370) was significantly greater than the RUIE (*M* = .025) (*t* = 13.432, *p* < .001). The RCIE of female respondents was significantly greater than that of male respondents (*p* < .01), whereas the RUIE did not significantly differ for any demographic variable.

In terms of marriage values, we found that the marriage utility value (*M* = 3.667) was notably greater than the marriage cost value (*M* = 3.177) (*t* = 26.222, *p* < .001). Specifically, males (*p* < .001), individuals from the eastern region (*p* < .01) and those from two-parent families (*p* < .01) reported higher marriage utility values. Conversely, males (*p* < .001) and individuals from the eastern (*p* < .01) region reported lower marriage cost values.

In terms of marriage expectations, 1261 Gen Z youths (91%) were willing to marry. Compared with males, females exhibited significantly stronger marriage intention (χ² = 30.783, *p* < .001). Generation Z individuals with a high school education or below anticipated earlier marriage (*F* = 4.749, *p* < .001).

### 4.3 Correlation analysis

After controlling for variables such as gender, family status, region, residence of family, and educational level, the various types of marriage values and online and offline exposure to marriage information among Gen Z in China were subjected to biased correlation analyses, as shown in S1 Table, S2 Table presents the bias correlations among the variables in the sample with marriage intention.

According to S1 Table, OEMU was significantly and positively correlated with the marriage utility values (*r* = .195, *p* < .01), and OEMC was significantly correlated with the marriage cost values (*r* = .236, *p* < .01) of Chinese Gen Z.

According to S2 Table, neither OEMU nor OEMC was significantly correlated with EMA. MUV was significantly negatively correlated with EMA (*r*=−.222, *p* < .01), and MCV was significantly positively correlated with EMA (*r* = .192, *p* < .01).

### 4.4 Regression analysis

The regression analyses of the effects of different types of online exposure to marriage information on marriage intention and expected marriage age are shown in S3 and S4 Tables. As shown in S3 Table, OEMU had a significant positive effect on Chinese Gen Z’s marriage intention (*β* = .278, *p* < .001), verifying Hypothesis 1a, whereas OEMU-Em had no significant effect on marriage intention. OEMC had a significant negative effect on Chinese Gen Z’s marriage intention (*β*=−.201, *p* < .001), verifying Hypothesis 1c. However, OEMC-Ps and OEMC-Ec had no significant effect on marriage intention. With respect to expected marriage age, according to S4 Table, OEMU and OEMC had no significant effect on expected marriage age; thus, Hypotheses 1b and 1d are not supported.

### 4.5 Mediation analysis

The results, as shown in Table 2, indicate that marriage values mediate the relationship between online exposure to marriage information and Chinese Gen Z’s marriage intention. Specifically, the mediating effect size of MUV was.224 (95% CI [.154,.309]), whereas that of MCV was − .329 (95% CI [−.438, − .238]). The data in Table 3 indicate that marriage values play an indirect role between online exposure to marriage information and expected marriage age. The indirect effect size of MUV was − .022 (95% CI [−.032, − .013]), whereas that of MCV was.026 (95% CI [.016,.037]). As neither confidence interval included zero, Hypotheses 2a–2d were supported. Crucially, regardless of the statistical significance of the impact of online exposure to marriage information on marriage expectations, the confidence intervals for the corresponding marriage value categories consistently excluded zero.

**Table 2:** Analysis of the mediating effect of different types of marriage values between online exposure to marriage information and marriage intention.

Effects	c	c’	a	b	ab	95% CI of ab
OEMU-Em → MUV-Em → MI	.124	.053	.056***	1.436***	.080	[.033,.137]
OEMU-S → MUV-S → MI	.163*	.120	.072***	1.202***	.087	[.042,.137]
OEMU-Ec → MUV-Ec → MI	.244***	.159*	.099***	1.025***	.102	[.067,.145]
OEMU-FC → MUV-FC → MI	.192**	.064	.134***	.936***	.125	[.086,.172]
**OEMU→ MUV → MI**	.278***	**.099**	.119***	1.878***	**.224**	**[.154,.309]**
OEMC-Ps → MCV-Ps → MI	−.095	.132	.103***	−1.669***	**−.172**	[**−**.250, **−**.107]
OEMC-O → MCV-O → MI	−.152*	.004	.123***	−.915 ***	−.113	[**−**.161, **−**.073]
OEMC-Ec → MCV-Ec → MI	−.026	.060	.156***	−.582***	−.091	[**−**.137, **−**.053]
OEMC-Py → MCV-Py → MI	−.277***	.022	.228***	−1.562***	−.356	[**−**.465, **−**.273]
**OEMC→ MCV → MI**	−.201***	**.157**	.174***	−1.890***	**−.329**	**[−.438, −.238]**

Notes: N = 1390. *p < .05, **p < .01, ***p < .001. c = total effect; c’ = direct effect; a = effect of the independent variable on the mediating variable; b = effect of the mediating variable on the dependent variable; ab = mediating effect; CI, confidence interval. MI, Marriage Intention; MUV, Marriage Utility Value; MCV, Marriage Cost Value; OEMU, Online Exposure to Marriage Utility information; OEMC, Online Exposure to Marriage Cost information; “A-B”, B type of A, e.g., “MUV-Em”, Marriage Emotional Utility Value; S, Security; Ec, Economic; FC, Family Continuity; Ps, Psychological; O, Opportunity; Py, Physiological.

**Table 3:** Analysis of the mediating effects of different types of marriage values between online exposure to marriage information and expected marriage age.

Effects	c	c’	a	b	ab	95% CI of ab
OEMU-Em → MUV-Em → EMA	−.002	.005	.036*	−.183***	−.007	[−.013, −.001]
OEMU-S → MUV-S → EMA	−.001	.009	.057**	−.164***	−.009	[−.016, −.003]
OEMU-Ec → MUV-Ec → EMA	−.040**	−.030*	.091***	−.105***	−.010	[−.016, −.005]
OEMU-FC → MUV-FC → EMA	−.027*	−.012	.116***	−.133***	−.015	[−.023, −.009]
**OEMU→ MUV → EMA**	**−.026**	**−.004**	.095***	−.231***	**−.022**	**[−.032, −.013]**
OEMC-Ps → MCV-Ps → EMA	.012	.001	.092***	.123***	.011	[.006,.018]
OEMC-O → MCV-O → EMA	.015	.000	.103***	.143***	.015	[.008,.023]
OEMC-Ec → MCV-Ec → EMA	.010	.002	.158***	.052***	.008	[.003,.015]
OEMC-Py → MCV-Py → EMA	.019	−.006	.218***	.116***	.025	[.016,.035]
**OEMC→ MCV → EMA**	**.022**	**−.004**	.161***	.160***	**.026**	**[.016,.037]**

Notes: N = 1261. *p < .05, **p < .01, ***p < .001. c = total effect; c’ = direct effect; a = effect of the independent variable on the mediating variable; b = effect of the mediating variable on the dependent variable; ab = mediating effect; CI, confidence interval. EMA, Expected Marriage Age; MUV, Marriage Utility Value; MCV, Marriage Cost Value; OEMU, Online Exposure to Marriage Utility information; OEMC, Online Exposure to Marriage Cost information; “A-B”, B type of A, e.g., “MUV-Em”, Marriage Emotional Utility Value; S, Security; Ec, Economic; FC, Family Continuity; Ps, Psychological; O, Opportunity; Py, Physiological.

### 4.6 Moderated mediation analysis

**Fig 2:**
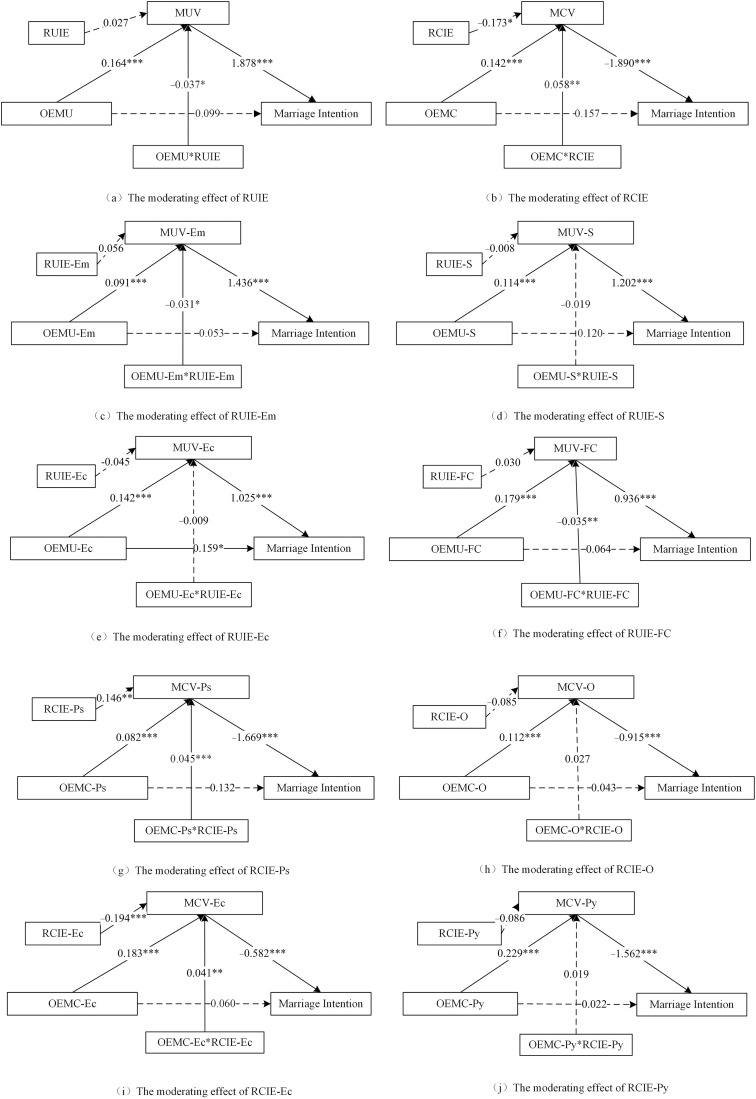
Analysis of moderated mediation effects: the influence of RUIE and RCIE by type. Notes: N = 1390. *p < .05, **p < .01, ***p < .001. MUV = Marriage Utility Value; OEMU = Online Exposure to Marriage Utility information; OEMC = Online Exposure to Marriage Cost information. MCV = Marriage Cost Value; Relative Utility Information Exposure; RCIE = Relative Cost Information Exposure; “A-B” represents B type of A, e.g., MUV-Em = Marriage Emotional Utility Value; Ec = Economic; FC = Family Continuity; Ps = Psychological; Py = Physiological; Em = Emotional; O = Opportunity; S = Security.

First, we examined the moderating effect of relative information exposure across the full sample. As shown in Fig 2, the interaction term between RUIE and OEMU was significantly negative, indicating that RUIE negatively moderated the relationship between OEMU and marriage utility values; thus, Hypothesis 4a was supported. Furthermore, relative emotional utility information exposure and online family continuity utility information negatively moderated the relationship between corresponding types of online exposure to marriage information and marriage utility values. However, the interaction term between RCIE and OEMC was significantly positive, indicating that RCIE positively moderated the relationship between OEMC and marriage cost value; thus, Hypothesis 3a was tested. Furthermore, relative psychological utility information exposure and economic cost information exposure negatively moderated the relationship between corresponding types of OEMC and marriage cost values.

**Fig 3:**
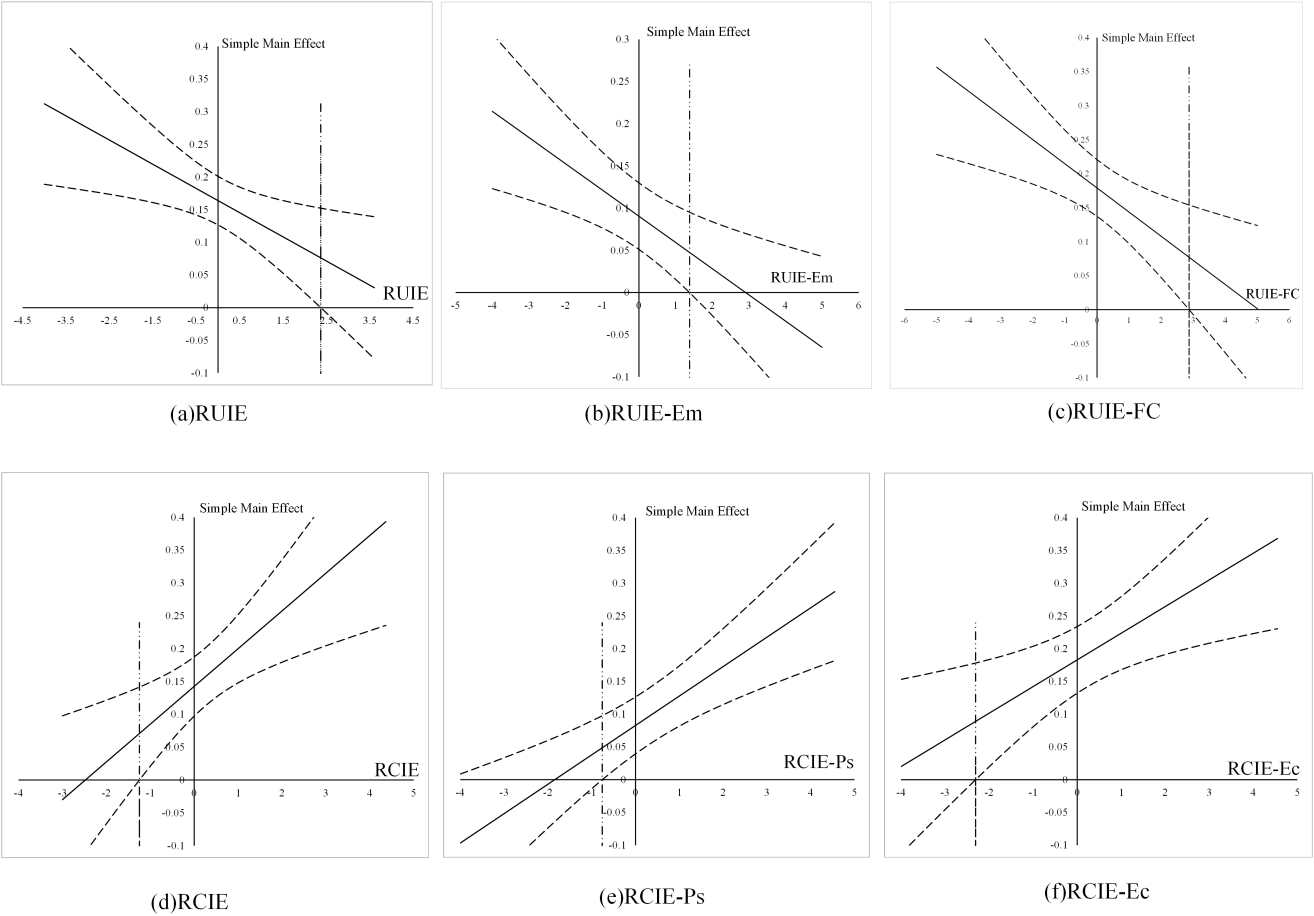
Unstandardized effects of internet information exposure on marriage values across various types and levels of relative information exposure. Notes: The dashed lines indicate the upper and lower bounds of the 95% confidence interval, and the vertical dashed line indicates the critical point where the confidence interval includes 0. The solid line shows the estimated effect. Relative Utility Information Exposure; RCIE = Relative Cost Information Exposure; “A-B” = B type of A, e.g., “RUIE-Em” = Relative Emotional Utility Information Exposure; Ec = Economic; FC = Family Continuity; Ps = Psychological; Em = Emotional.

J‒N plots were constructed to investigate the specific shape of the moderating effect. As shown in Fig 3, as RUIE, including emotional utility and family continuity utility, increased, the impact of OEMU on the corresponding marriage utility value gradually decreased and eventually disappeared. Conversely, as RCIE, including psychological and economic costs, increased, the effect of OEMC on the corresponding marriage cost value transitioned from nonsignificant to significant and then gradually increased.

As shown in Table 4, the mediating effect of the marriage cost value for Gen Z in China was − .213 (95% CI [−.335, − .104]) at lower levels of RCIE. Conversely, at higher levels of RCIE, the mediating effect value of cost values was − .406 (95% CI [−.560, − .283]). These data suggest that the mediating role of cost values between OEMC and marriage intention is positively moderated by RCIE, thereby supporting Hypothesis 3b. Additionally, relative psychological and economic cost information exposure positively moderated the mediating role of the corresponding types of cost values. Conversely, at lower levels of RUIE, the mediating effect value of marriage value for Gen Z in China was.361 (95% CI [.257,.495]). At higher levels of RUIE, the mediating effect of marriage utility values was.239 (95% CI [.138,.356]). This finding indicates that the mediating role of marriage utility values in the relationship between OEMU and marriage intention is negatively moderated by RUIE, thus verifying Hypothesis 4b. Furthermore, relative emotional utility and family continuity utility information exposure negatively moderated the mediating role of the corresponding types of utility values.

**Table 4:** Mediating effects of various types of marriage values at different levels of their corresponding relative information exposure (N = 1390).

Moderating variable	Mediating effect	BootSE	95% CI
Mediating Effects of MUV-Em at Different Levels of RUIE-Em			
M − SD	.174	.039	[.104,.259]
M	.130	.033	[.071,.199]
M + SD	.086	.036	[.019,.158]
Mediating Effects of MUV-FC at Different Levels of RUIE-FC			
M − SD	.201	.034	[.140,.271]
M	.168	.027	[.119,.225]
M + SD	.135	.027	[.085,.193]
**Mediating Effects of UV at Different Levels of RUIE**			
M − SD	.361	.061	[.257,.495]
M	.309	.048	[.225,.416]
M + SD	.239	.056	[.138,.356]
Mediating Effects of MCV-Ps at Different Levels of RCIE-Ps			
M − SD	−.062	.046	[−.158,.028]
M	−.137	.041	[−.224, −.062]
M + SD	−.289	.060	[−.415, −.182]
Mediating Effects of MCV-Ec at Different Levels of RCIE-Ec			
M − SD	−.083	.024	[−.137, −.041]
M	−.106	.025	[−.163, −.062]
M + SD	−.130	.030	[−.196, −.077]
**Mediating Effects of MCV at Different Levels of RCIE**			
M − SD	−.213	.059	[−.335, −.104]
M	−.296	.056	[−.415, −.193]
M + SD	−.406	.072	[−.560, −.283]

Notes: CI, confidence interval. MUV, Marriage Utility Value; MCV, Marriage Cost Value; RUIE, Relative Utility Information Exposure; RCIE, Relative Cost Information Exposure; “A-B”, “B type of A”, e.g., “MUV-Em”, Marriage Emotion Utility Value; Ec, Economic; FC, Family Continuity; Ps, Psychological.

Subsequently, to examine Hypothesis 3c and Hypothesis 4c, we further investigated the moderating effect of relative information exposure within the subgroup sample of participants with marriage intentions, as illustrated in S1 Fig. Within the sample of individuals with marriage intentions, RCIE moderated the relationship between OEMC and MCV, whereas RUIE did not moderate the relationship between OEMU and MUV (S1 Fig). The J‒N plot in S2 Fig also further confirms these results. We further performed a moderated mediation analysis to examine Hypothesis 3c and Hypothesis 4c, with the results shown in Table 5. The mediating effect of marriage value for Gen Z in China was.018 (95% CI [.008,.029]) at lower levels of RCIE. Conversely, at higher levels of RCIE, the mediating effect value of cost values was.029 (95% CI [.018,.044]). Relative psychological and economic cost information exposure positively moderated the mediating role of the corresponding types of cost values. Therefore, Hypothesis 3c was supported, but Hypothesis 4c was not.

**Table 5:** Mediating effects of various types of marriage values at different levels of their corresponding relative information exposure (N = 1261).

Moderating variable	Mediating effect	BootSE	95% CI
Mediating Effects of MCV-Ps at Different Levels of RCIE-Ps			
M − SD	.004	.004	[−.004,.011]
M	.010	.003	[.004,.018]
M + SD	.017	.005	[.009,.027]
Mediating Effects of MCV-Ec at Different Levels of RCIE-Ec			
M − SD	.008	.003	[.002,.015]
M	.010	.004	[.004,.018]
M + SD	.013	.005	[.004,.023]
**Mediating Effects of MCV at Different Levels of RCIE**			
M − SD	.018	.006	[.008,.029]
M	.023	.006	[.013,.035]
M + SD	.029	.007	[.018,.044]

Notes: CI, Confidence Interval. MCV, Marriage Cost Value; RCIE, Relative Cost Information Exposure; MCV-Ps, Marriage Psychological Cost Value; MCV-Ec, Marriage Economic Cost Value.

## 5 Discussion

This study investigated the mechanisms by which online exposure to marriage information and RIE influence the marriage values and marriage expectations of Chinese Gen Z. The research focused on how these factors shape the marriage expectations of Gen Z. The findings confirm that online exposure to marriage information impacts Chinese Gen Z’s marriage expectations through their marriage values. Additionally, different types of online exposure to marriage information, marriage values, and marriage expectations form a mediation model with varying effects. RUIE and RCIE play opposite moderating roles in this relationship.

### 5.1 Association between online exposure to marriage information, marriage value, and marriage expectations

An intriguing finding of this study is that online exposure to marriage information has a statistically significant influence on marriage intention among Gen Z youth, whereas its impact on their expected marriage age remains negligible. Exposure to such information enables individuals to directly perceive the utility and costs associated with marriage, thereby reshaping Gen Z’s value judgments and behavioral inclinations toward matrimony. Notably, exposure to content emphasizing practical utilities—including social security benefits, economic advantages, and family continuity—positively enhances marriage intention, whereas emotion-centric information has no such effect. A plausible explanation lies in Gen Z’s diversified avenues for fulfilling emotional needs (e.g., nonmarriage romantic relationships), reducing dependence on marriage as the sole pathway for emotional gratification. In contrast, EMA appears to be predominantly shaped by individualized life trajectories [6], including career planning, financial readiness, and academic studies—factors that remain largely impervious to online information. Subsequent analysis further reveals that EMA becomes susceptible to influence only when Gen Z actively internalizes and cognitively processes such information to form enduring value judgments about marriage.

In examining the relationships between marriage utility values and marriage intention/expected marriage age, emotional and security-oriented values demonstrated stronger predictive effects, whereas economic and family continuity values exhibited weaker predictive power. With respect to the association between marriage cost values and marriage intention, psychological and physiological cost values exerted the most substantial negative influence. This aligns with recent research indicating that Gen Z prioritizes the emotional and spiritual aspects of marriage [16]. Similarly, the potential emotional and psychological costs and threats of marriage have greater degrees of suppression and impact on the marriage expectations of Gen Z. In terms of the correlations between utility values and marriage expectations, Chinese Gen Z places greater emphasis on achieving personal goals and values within marriage, with a focus on shaping intimate relationships. Marriage and family serve as vehicles for expressing personal emotions and feelings, with a strong emphasis on fulfilling emotional needs over family continuity [43]. In the context of China’s rapid economic development and the improvement of the social security system, the state provides reliable protection for the physical and emotional security of its citizens. Consequently, Gen Z youth, born and raised during this period, have idealized marriage values distinct from those of other generations [16]. They prioritize individual spiritual and emotional satisfaction, rendering economic utility values less influential on their marriage expectations. For instance, most college students believe that marriage is not merely an exchange of benefits [16]. In terms of the correlations between cost values and marriage expectations, current Chinese Gen Z marriages are shifting toward individual experiences, emphasizing the self-experience of marriage and marital life [44]. Psychological and physiological costs had the strongest negative impact on marriage expectations because of their direct effect on the individual. Conversely, economic costs had the weakest negative effect on marriage expectations among China’s Gen Z. In the context of China’s rapid economic and social development, the state provides comprehensive social security support, enabling Gen Z to pursue true love and emotional fulfillment. As a result, Gen Z is more open to various forms of marriage that incur lower financial costs, such as naked marriages and traveling marriages. Another critical point that should not be overlooked is that opportunity cost values have the strongest influence on the expected age of marriage, as Generation Z is more likely to delay marriage in the pursuit of higher-quality partners and to prioritize the achievement of personal career or educational goals [45].

### 5.2 The mediation effect of marriage value

The results of the mediation analysis reveal that OEMU positively influences the marriage intention of Gen Z in China, with marriage utility values playing a mediating role, whereas OEMC negatively influences the marriage intention of Gen Z, with marriage cost values playing a mediating role. Furthermore, the results of this study indicate that marriage values exhibit statistically significant indirect effects on the relationship between exposure to online marriage information and expected marriage age.

The findings indicate that marriage utility values and marriage cost values mediate the effects of OEMU and OEMC on marriage intention and the expected marriage age of Gen Z. Regardless of whether or not a particular type of online exposure has a significant effect on marriage intention and expected marriage age, that type of online exposure influences and shapes the corresponding type of marriage value, which in turn affects marriage intention and expected marriage age. One of the marginal contributions of this study, compared with previous research, is that it clarifies the mediating mechanism underlying the association between online exposure to marriage information and marriage intention and expected marriage age. The literature, which is usually based on the socialization theory perspective, suggests that the internet provides online socialization paths for Gen Z and that the level of online participation of Gen Z youth negatively affects their marriage intention [9]. This study further focuses on the frequency of online exposure to the marriage information of Gen Z in China, refines the online marriage information into eight types, verifies the mediating role of each type of marriage value in the relationship between each type of online exposure and marriage intention based on the cultivation theory, and explores the black-box mechanism between the two. In addition, Gen Z in China prefers to obtain marriage information from online sources and form corresponding cognitions based on the various types of marriage information obtained. The social interaction between individuals and online information enhances their knowledge, understanding, and value judgment of marriage; strengthens their corresponding types of marriage values; and thus strengthens or weakens their attitudes and intentions toward marriage [12]. When the fragmented and sensitive marriage values of Gen Z are influenced by the fragmented cost information on the internet, they are more likely to develop extremely negative and high-cost values for marriage, which may lead to the phenomenon of “fear of marriage”.

### 5.3 The moderating effect of relative information exposure

Moderating effects analyses in the full sample revealed that RUIE negatively moderated the effect of OEMU on marriage utility values and the mediating role of marriage utility values, whereas RCIE positively moderated the effect of OEMC on marriage cost values and the mediating role of marriage cost values. However, the ultimate impacts of both were the same. Compared with obtaining marriage information from offline sources, when the participants had high RCIE and RUIE, the impact of OEMU on marriage utility values was diminished, and the impact of OEMC on marriage cost values was enhanced, which ultimately reduced the marriage intention of Chinese Gen Z. However, in the sample with marriage intentions, the moderating effect of RUIE was not validated. When individuals had marriage intentions, RCIE enhanced the positive influence of OEMC on marriage cost values, whereas the indirect effect of exposure to online marriage cost information on expected marriage age increased, thereby prolonging individuals’ expected marriage age. From another perspective, RCIE not only suppresses marriage intentions but also delays marriage.

This study argues that the moderating effect of RIE arises from a discrepancy between the online portrayal of marriage utility and costs and the actual utility and costs of marriage. This discrepancy includes both the objective distortion of marriage-related information and the subjective biases in how China’s Gen Z interprets these messages. With respect to online marriage utility information, China’s Gen Z exhibits romantic and idealistic visions of marriage. Celebrity marriages and portrayals of perfect partners increase their expectations for their own marriages. However, the real marriage market often fails to meet these heightened expectations. Additionally, excessive online exposure to marriage information can lead to information overload. This overload weakens their ability to interpret the information accurately, thereby diminishing the marginal impact of online marriage utility information on their utility values. Consequently, the relative exposure of China’s Gen Z to learn about marriage utility from the internet leads to a rift between online marriage utility information and the actual utility of marriage. Chinese Gen Z demonstrates deficiencies in effectively interpreting and processing online marriage utility information. Consequently, OEMU has a diminished impact on their marriage utility values, which weakens their marriage intention. In contrast, online marriage cost information is often exaggerated by the media to attract attention. This exaggeration leads to overinterpretation and endless debates among different circles and subgroups of China’s Gen Z. It can even provoke gender antagonisms and exaggerate the perceived costs and risks of marriage [16]. Even when realistic information about the cost of marriage is disseminated, the role of the internet and social media may lead to the social mobilization phenomenon of risk amplification and circle spread, which in turn spreads the “fear of marriage and childbearing” sentiment [33]. As a result, when China’s Gen Z prefers to learn about marriage costs from the internet, they often misinterpret and overestimate these costs. This disconnect between online information and the actual cost of marriage leads to a higher perceived value of marriage costs, thereby weakening their marriage intentions.

### 5.4 Implications

This study offers several theoretical contributions. First, it examines the effects of different types of online exposure to marriage information on marriage expectations, thereby enriching our knowledge of the antecedent variables of marriage expectations. Compared with previous research, this study aligns with the characteristics of the digital age and China’s Gen Z, refining the impact of individuals’ exposure to marriage information on marriage expectations. Second, it explores the mediating role of marriage values in the relationship between online exposure to marriage information and marriage expectations. This extends the application of cultivation theory in the field of new media and enhances the theoretical community’s understanding of how online exposure to marriage information influences marriage expectations. This comparison helps clarify the unique attitudes of China’s Gen Z toward marriage. Third, by examining the moderating role of relative information exposure, this study clarifies the boundary conditions for the formation of marriage values, providing new insights into the relationship between online and offline socialization among Chinese Gen Z.

On the practical side, the results of this study reveal that different types of online marriage information can shape corresponding marriage values, thereby influencing the marriage expectations of China’s Gen Z. Therefore, strengthening the supervision and guidance of online media and creating a positive and objective online environment for the acquisition of marriage information, including the examination and standardization of community conventions and rules on all online platforms, are crucial. Establishing formal and authoritative platforms for disseminating scientific information and debunking rumors is essential. Furthermore, three categories of content require enhanced regulatory scrutiny and corrective measures: (1) commercialized fertility interventions, (2) the dissemination of misinformation regarding reproductive health, particularly the distortion of matrimonial norms, and (3) malicious advocacy promoting demographic destabilization. Various channels can be utilized to share positive stories about Chinese marriage and love, moderately supplementing the publicity of traditional family functions of marriage, such as family continuity, to inspire Gen Z’s aspirations for a better married life.

Moreover, strengthening the protection of the utility of marriage and reducing the potential costs of marriage through systematic policy and institutional design are necessary. This can promote the formation of marriage utility values and inhibit the formation of marriage cost values among Gen Z, thereby increasing marriage expectations. For instance, improving and implementing systems for marriage leave, maternity leave, and paternity leave; accelerating the improvement of legislation related to marriage and childbirth, such as the punishment and education of psychological (mental) violence; and promoting the reform of marriage customs to advocate new and simple marriage customs are essential steps. Given that China’s Gen Z prefers to obtain information from online channels, it is crucial to strategically develop their online socialization and coordinate and integrate online and offline socialization. When coordinating and integrating online and offline socialization, attention should be given to the relationships between the whole and the parts, emphasizing the promotion of offline marriage information to weaken the inhibitory effect of relative information exposure on marriage expectations.

### 5.5 Limitations and future research

However, this study has several limitations. First, the cross-sectional design precluded rigorous testing of causal relationships between the variables. Future research could address this by employing longitudinal or experimental designs. Second, the reliance on self-reported responses from Chinese Gen Z youth introduced potential biases, despite statistical tests indicating no serious common methodological bias; there may still be various noises that interfere with the transmission of information in the contact between online and offline marriage information. Given the increasing diversity of information types, respondents may encounter difficulties in accurately recalling and reporting their exposure to a specific category of information [46]. To increase the accuracy of the conclusions of this study, future research could adopt a multisource design and more effective measurement of exposure to marriage information, e.g., passive measurement. Third, although this study focuses on how online exposure to marriage information affects marriage expectations, we have not fully explored whether such exposure influences individuals’ love values and romantic attitudes. We therefore recommend future research to investigate how online information exposure shapes romantic relationships. Although the study systematically collected questionnaires from Gen Z individuals across 30 provinces and cities in China, whether the relationships between variables differ across subgroups and genders merits further in-depth investigation. Finally, given that our measurement of expected marriage age employed categorical age ranges rather than precise single-year estimates, this approach may have resulted in a less precise treatment of anticipated marriage timing. Therefore, future studies should adopt specific expected marriage ages to validate the conclusions of this study.

## 6 Conclusion

This study explored the mechanisms by which different types of online exposure to marriage information affect Chinese Gen Z’s marriage expectations from the perspective of their processing and handling of online marriage information. With reference to cultivation theory, this study revealed that regardless of the correlation between a given type of online exposure to marriage information and marriage expectations, online exposure to marriage information reinforces that type of marriage value, which in turn impacts Gen Z’s marriage intention and expected marriage age. In addition, when a Gen Z individual has high RUIE and RCIE, the OEMU has a weaker effect on marriage utility values, whereas the OEMC has a stronger effect on marriage cost values and lower marriage intention. Therefore, improving the form and content of online marriage information and optimizing the RIE and marriage values of Chinese Gen Z are crucial for increasing their marriage expectations.

## Supporting information

S1 Fig Analysis of moderated mediation effects: the influence of RUIE and RCIE by type. Notes: N = 1261; *p < .05, **p < .01, ***p < .001. EMA = Expected Marriage Age; MUV = Marriage Utility Value; OEMU = Online Exposure to Marriage Utility information; OEMC = Online Exposure to Marriage Cost information. MCV = Marriage Cost Value; Relative Utility Information Exposure; RCIE = Relative Cost Information Exposure; “A-B” represents B type of A, e.g., MUV-Em = Marriage Emotional Utility Value; Ec = Economic; FC = Family Continuity; Ps = Psychological; Py = Physiological; Em = Emotional; O = Opportunity; S = Security.
(TIF)

S2 Fig Unstandardized effects of online information exposure on marriage values across various types and levels of relative information exposure. Notes: The dashed lines indicate the upper and lower bounds of the 95% confidence interval, while the vertical dashed line marks the critical point where the confidence interval includes 0. The solid line shows the estimated effect. RCIE = Relative Cost Information Exposure; RCIE-Ps = Relative Psychological Cost Information Exposure; RCIE-Ec = Relative Economic Cost Information Exposure.
(TIF)

S1 Table Bias correlation analysis in the full sample. Notes: N = 1390, *p < .05, **p < .01. MUV = Marriage Utility Value; MCV = Marriage Cost Value; OEMU = Online Exposure to Marriage Utility information; OEMC = Online Exposure to Marriage Cost information; OfEMU = Offline Exposure to Marriage Utility information; OfEMC = Offline Exposure to Marriage Cost information; RUIE = Relative Utility Information Exposure; RCIE = Relative Cost Information Exposure; “A-B” = B type of A, e.g., “MUV-Em” = Marriage Emotional Utility Value; S = Security; Ec = Economic; FC = Family Continuity; Ps = Psychological; O = Opportunity; Py = Physiological.
(PDF)

S2 Table Bias correlation analysis in the sample with marriage intention. Notes: N = 1261, *p < .05, **p < .01. EMA = Expected Marriage Age; MUV = Marriage Utility Value; MCV = Marriage Cost Value; OEMU = Online Exposure to Marriage Utility information; OEMC = Online Exposure to Marriage Cost information; OfEMU = Offline Exposure to Marriage Utility information; OfEMC = Offline Exposure to Marriage Cost information; RUIE = Relative Utility Information Exposure; RCIE = Relative Cost Information Exposure; “A-B” = B type of A, e.g., “MUV-Em” = Marriage Emotional Utility Value; S = Security; Ec = Economic; FC = Family Continuity; Ps = Psychological; O = Opportunity; Py = Physiological.
(PDF)

S3 Table The regression results of online exposure to marriage information, marriage value and marriage intention. Notes: The value in parentheses is Exp(B). N = 1390. *p < .05, **p < .01, ***p < .001. MI = Marital Intention; MUV = Marriage Utility Value; MCV = Marriage Cost Value; OEMU = Online Exposure to Marriage Utility information; OEMC = Online Exposure to Marriage Cost information; “A-B” = B type of A, e.g., “MUV-Em” = Marriage Emotional Utility Value; S = Security; Ec = Economic; FC = Family Continuity; Ps = Psychological; O = Opportunity; Py = Physiological.
(PDF)

S4 Table The regression results of online exposure to marriage information, marriage value and expected marriage age. Notes: N = 1261. *p < .05, **p < .01, ***p < .001. EMA = Expected Marriage Age; MUV = Marriage Utility Value; MCV = Marriage Cost Value; OEMU = Online Exposure to Marriage Utility information; OEMC = Online Exposure to Marriage Cost information; “A-B” = B type of A, e.g., “MUV-Em” = Marriage Emotional Utility Value; S = Security; Ec = Economic; FC = Family Continuity; Ps = Psychological; O = Opportunity; Py = Physiological.
(PDF)

S1 File Original data. (XLSX)

S2 File Scales used in this study. (DOCX)

## Abbreviations

ME: marriage expectation
OEMU: online exposure to marriage utility information
OEMC: online exposure to marriage cost information
RIE: relative information exposure
RUIE: relative utility information exposure
RCIE: relative cost information exposure
Gen Z: generation Z
