# Supplementary figures and images for: Online exposure to marriage information and marriage expectations of Generation Z in China: The roles of marriage value and relative information exposure
Source: PLoS One. 2025 Oct 27;20(10):e0334596. doi: 10.1371/journal.pone.0334596 (PMC12558505; doi:10.1371/journal.pone.0334596)

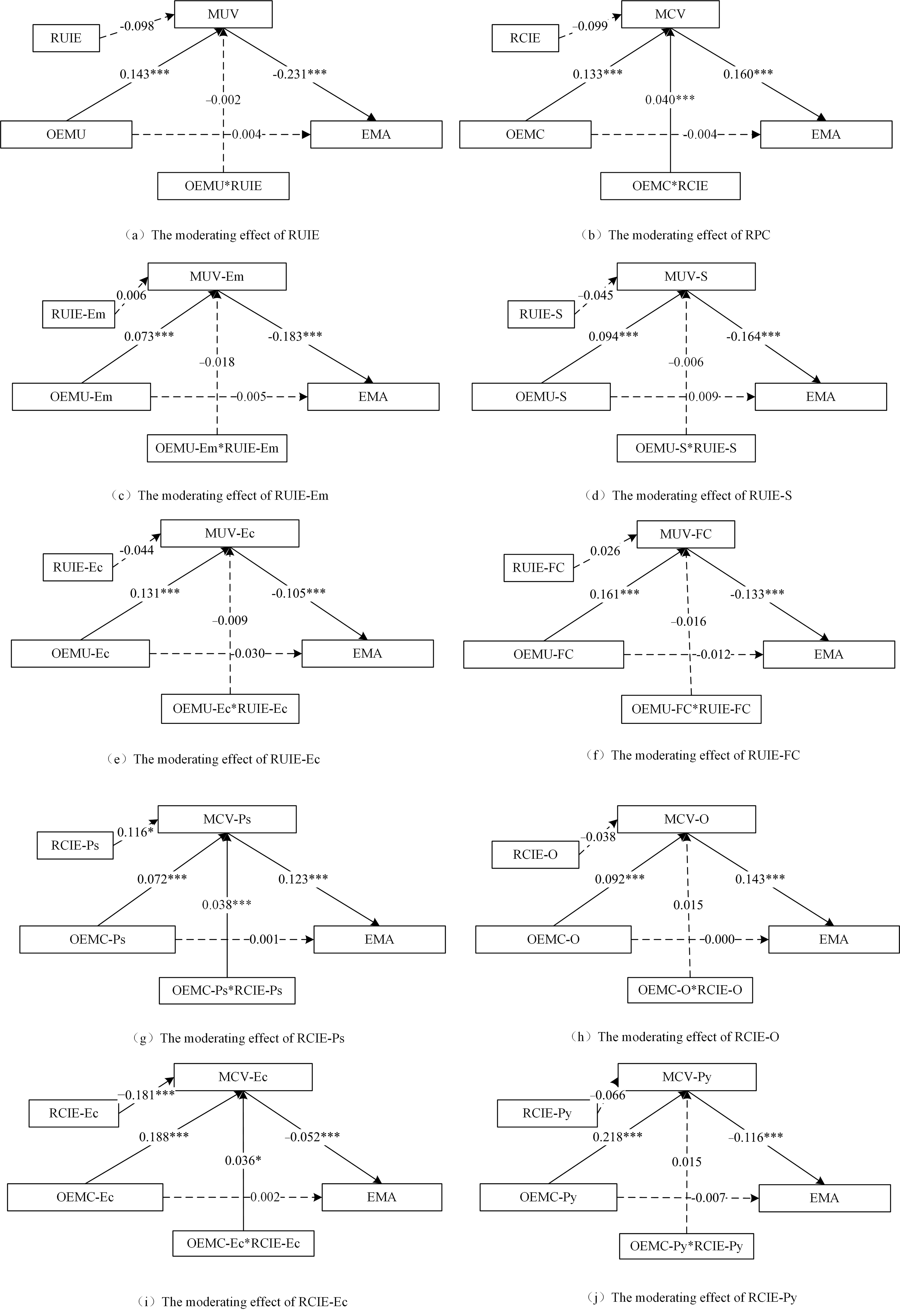

Supplement: S1 Fig — Notes: N = 1261; *p < .05, **p < .01, ***p < .001. EMA = Expected Marriage Age; MUV = Marriage Utility Value; OEMU = Online Exposure to Marriage Utility information; OEMC = Online Exposure to Marriage Cost information. MCV = Marriage Cost Value; Relative Utility Information Exposure; RCIE = Relative Cost Information Exposure; “A-B” represents B type of A, e.g., MUV-Em = Marriage Emotional Utility Value; Ec = Economic; FC = Family Continuity; Ps = Psychological; Py = Physiological; Em = Emotional; O = Opportunity; S = Security. (TIF) [file pone.0334596.s001.tif]

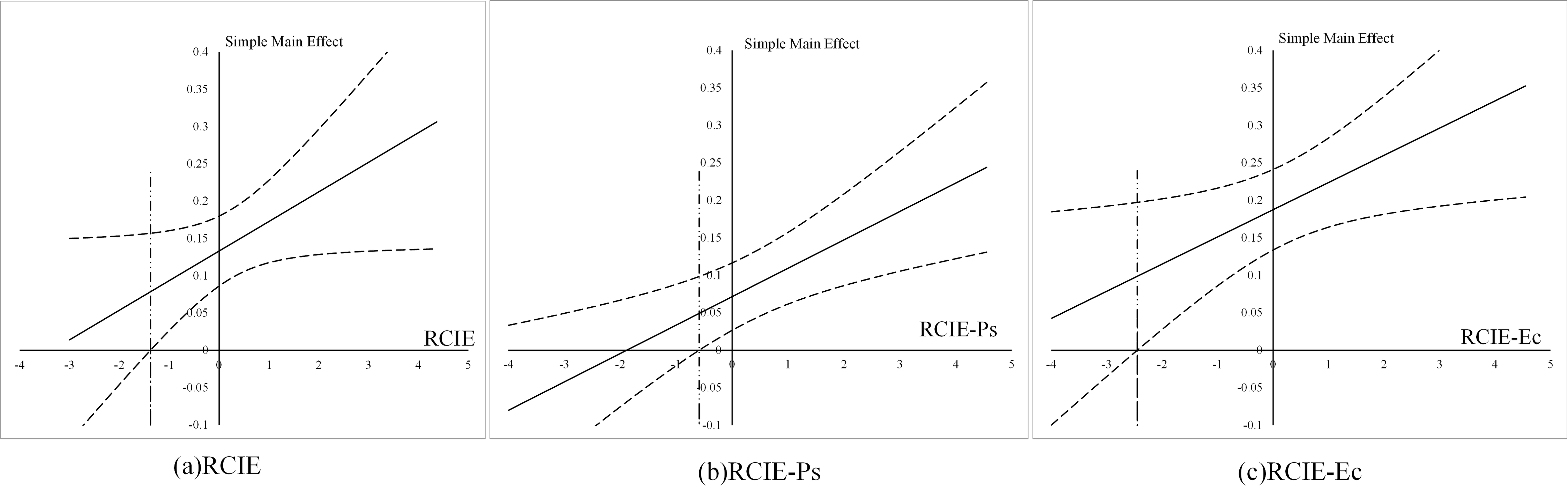

Supplement: S2 Fig — Notes: The dashed lines indicate the upper and lower bounds of the 95% confidence interval, while the vertical dashed line marks the critical point where the confidence interval includes 0. The solid line shows the estimated effect. RCIE = Relative Cost Information Exposure; RCIE-Ps = Relative Psychological Cost Information Exposure; RCIE-Ec = Relative Economic Cost Information Exposure. (TIF) [file pone.0334596.s002.tif]
