# Supplementary material for: Online exposure to marriage information and marriage expectations of Generation Z in China: The roles of marriage value and relative information exposure
Source: PLoS One. 2025 Oct 27;20(10):e0334596. doi: 10.1371/journal.pone.0334596 (PMC12558505; doi:10.1371/journal.pone.0334596)
Supplement: S1 Table — Notes: N = 1390, *p < .05, **p < .01. MUV = Marriage Utility Value; MCV = Marriage Cost Value; OEMU = Online Exposure to Marriage Utility information; OEMC = Online Exposure to Marriage Cost information; OfEMU = Offline Exposure to Marriage Utility information; OfEMC = Offline Exposure to Marriage Cost information; RUIE = Relative Utility Information Exposure; RCIE = Relative Cost Information Exposure; “A-B” = B type of A, e.g., “MUV-Em” = Marriage Emotional Utility Value; S = Security; Ec = Economic; FC = Family Continuity; Ps = Psychological; O = Opportunity; Py = Physiological. (PDF) [file pone.0334596.s003.pdf]

S1 Table. Bias correlation analysis in the full sample.

	M	SD	1	a	b	c	d	2	e	f	g	h	3	4	5	6	7	8
1.MUV	3.667	.697	1															
a.MUV-Em	3.795	.810	.839**	1														
b.MUV-S	3.490	.889	.851**	.621**	1													
c.MUV-Ec	3.678	.761	.790**	.511**	.551**	1												
d.MUV-FC	3.724	.921	.786**	.592**	.561**	.510**	1											
2.MCV	3.177	.807	−.502**	−.420**	−.430**	−.414**	−.371**	1										
e.MCV-Ps	3.203	.912	−.457**	−.399**	−.389**	−.362**	−.336**	.821**	1									
f.MCV-O	2.709	.957	−.437**	−.355**	−.373**	−.369**	−.326**	.812**	.583**	1								
g.MCV-Ec	3.458	1.010	−.255**	−.205**	−.215**	−.233**	−.175**	.788**	.492**	.489**	1							
h.MCV-Py	3.338	1.033	−.511**	−.429**	−.445**	−.403**	−.389**	.864**	.652**	.609**	.572**	1						
3.OEMU	2.987	1.081	.195**	.157**	.152**	.154**	.186**	−.082**	−.078**	.037	.046	−.109**	1					
OEMU-Em	3.344	1.313	.112**	.095**	.078**	.076**	.126**	.032	.028	.002	.030	.047	.822**					
OEMU-S	2.946	1.287	.143**	.117**	.112**	.112**	.133**	.043	.041	.012	.018	−.070**	.853**					
OEMU-Ec	2.935	1.304	.185**	.140**	.142**	.174**	.152**	−.098**	−.097**	−.055*	.051	−.121**	.833**					
OEMU-FC	2.724	1.340	.206**	.164**	.168**	.147**	.203**	−.097**	−.090**	−.056*	−.053*	−.121**	.797**					
4.OEMC	3.204	1.072	−.081**	−.085**	−.072**	−.056*	.046	.236**	.186**	.197**	.174**	.217**	.628**	1				
OEMC-Ps	3.225	1.339	−.030	.037	.039	.010	.005	.150**	.153**	.118**	.101**	.122**	.551**	.820**				
OEMC-O	3.091	1.308	−.030	.040	.024	.012	.019	.150**	.107**	.173**	.093**	.123**	.567**	.823**				
OEMC-Ec	3.121	1.257	−.025	.028	.001	.046	.003	.188**	.132**	.132**	.195**	.153**	.514**	.788**				
OEMC-Py	3.380	1.357	−.177**	−.168**	−.166**	−.114**	−.122**	.278**	.211**	.217**	.178**	.307**	.408**	.815**				
5.OfMUE	2.962	.989	.202**	.135**	.171**	.171**	.190**	−.094**	−.099**	−.069*	.039	−.105**	.568**	.482**	1			
6.OfMCE	2.834	.985	.023	.010	.020	.026	.050	.102**	.044	.076**	.115**	.096**	.435**	.570**	.647**	1		
7.RUIE	.025	.964	.012	.037	.005	.002	.014	.004	.014	.029	.012	.014	.540**	.211**	−.386**	−.173**	1	
8.RCIE	.370	.958	−.115**	−.084**	−.100**	−.089**	−.104**	.155**	.161**	.140**	.074**	.141**	.245**	.518**	−.137**	−.408**	.415**	1

Notes: N= 1390, *p<.05, **p<.01. MUV = Marriage Utility Value; MCV = Marriage Cost Value; OEMU = Online Exposure to Marriage Utility information; OEMC = Online Exposure to Marriage Cost information; OfEMU = Offline Exposure to Marriage Utility information; OfEMC = Offline Exposure to Marriage Cost

information; RUIE = Relative Utility Information Exposure; RCIE = Relative Cost Information Exposure; “A-B” = B type of A, e.g., “MUV-Em” = Marriage Emotional Utility Value; S = Security; Ec = Economic; FC = Family Continuity; Ps = Psychological; O = Opportunity; Py = Physiological.
