# Supplementary material for: Online exposure to marriage information and marriage expectations of Generation Z in China: The roles of marriage value and relative information exposure
Source: PLoS One. 2025 Oct 27;20(10):e0334596. doi: 10.1371/journal.pone.0334596 (PMC12558505; doi:10.1371/journal.pone.0334596)
Supplement: S2 Table — Notes: N = 1261, *p < .05, **p < .01. EMA = Expected Marriage Age; MUV = Marriage Utility Value; MCV = Marriage Cost Value; OEMU = Online Exposure to Marriage Utility information; OEMC = Online Exposure to Marriage Cost information; OfEMU = Offline Exposure to Marriage Utility information; OfEMC = Offline Exposure to Marriage Cost information; RUIE = Relative Utility Information Exposure; RCIE = Relative Cost Information Exposure; “A-B” = B type of A, e.g., “MUV-Em” = Marriage Emotional Utility Value; S = Security; Ec = Economic; FC = Family Continuity; Ps = Psychological; O = Opportunity; Py = Physiological. (PDF) [file pone.0334596.s004.pdf]

S2 Table. Bias correlation analysis in the sample with marriage intention

	M	SD	1	2	a	b	c	d	3	e	f	g	h	4	5	6	7	8	9
1.EMA	1.278	0.629	1																
2.MUV	3.756	0.634	−.222**	1															
a.MUV-Em	3.900	0.716	−.200**	.807**	1														
b.MUV-S	3.586	0.838	−.203**	.847**	.579**	1													
c.MUV-Ec	3.742	0.727	−.130**	.779**	.462**	.534**	1												
d.MUV-FC	3.819	0.859	−.179**	.768**	.546**	.541**	.472**	1											
3.MCV	3.096	0.788	.192**	−.458**	−.371**	−.393**	−.369**	−.327**	1										
e.MCV-Ps	3.110	0.885	.170**	−.409**	−.349**	−.342**	−.319**	−.295**	.812**	1									
f.MCV-O	2.628	0.933	.208**	−.400**	−.313**	−.346**	−.326**	−.292**	.800**	.562**	1								
g.MCV-Ec	3.413	1.018	.085**	−.224**	−.176**	−.192**	−.201**	−.140**	.792**	.492**	.475**	1							
h.MCV-Py	3.235	1.012	.173**	−.473**	−.383**	−.412**	−.364**	−.350**	.857**	.629**	.589**	.570**	1						
4.OEMU	3.014	1.069	−0.045	.170**	.127**	.129**	.138**	.157**	−.073**	−.067*	−0.036	−0.041	−.096**	1					
OEMU-Em	3.358	1.295	−0.004	.085**	.067*	0.047	.064*	.105**	−0.028	−0.022	0.002	−0.029	−0.042	.817**					
OEMU-S	2.969	1.269	−0.002	.112**	.080**	.092**	.093**	.098**	−0.028	−0.027	−0.006	−0.010	−0.050	.847**					
OEMU-Ec	2.973	1.302	−.082**	.170**	.123**	.131**	.165**	.125**	−.088**	−.088**	−0.053	−0.044	−.106**	.830**					
OEMU-FC	2.758	1.335	−.057*	.189**	.148**	.151**	.130**	.187**	−.095**	−.082**	−.059*	−0.053	−.117**	.799**					
5.OEMC	3.173	1.050	0.035	−.070*	−.071*	−.068*	−0.034	−0.047	.218**	.167**	.171**	.165**	.207**	.639**	1				
OEMC-Ps	3.199	1.320	0.025	−0.040	−0.047	−0.050	−0.003	−0.025	.136**	.139**	.100**	.093**	.117**	.564**	.816**				
OEMC-O	3.061	1.290	0.031	−0.002	−0.017	−0.010	0.024	−0.003	.125**	.082**	.145**	.077**	.104**	.572**	.816**				
OEMC-Ec	3.114	1.247	0.020	−0.029	−0.030	−0.006	−0.044	−0.011	.187**	.134**	.120**	.195**	.154**	.510**	.784**				
OEMC-Py	3.320	1.335	0.039	−.153**	−.135**	−.153**	−.088**	−.112**	.257**	.182**	.188**	.171**	.294**	.415**	.810**				
6.OfMUE	2.987	0.983	−0.023	.211**	.138**	.170**	.182**	.191**	−.099**	−.100**	−.083**	−0.040	−.104**	.583**	.498**	1			
7.OfMCE	2.838	0.970	0.010	0.029	−0.019	0.030	0.038	0.047	.095**	0.047	0.051	.111**	.095**	.451**	.594**	.651**	1		
8.RUIE	0.027	0.936	−0.027	−0.027	0.001	−0.032	−0.034	−0.020	0.020	0.028	0.046	−0.005	−0.001	.528**	.207**	−.382**	−.168**	1	
9.RCIE	0.336	0.913	0.030	−.111**	−.061*	−.110**	−.080**	−.105**	.147**	.140**	.141**	.070*	.135**	.246**	.505**	−.130**	−.394**	.416**	1

Notes: N= 1261, **p*<.05, ***p*<.01. EMA = Expected Marriage Age; MUV = Marriage Utility Value; MCV = Marriage Cost Value; OEMU = Online Exposure to Marriage Utility information; OEMC = Online Exposure to Marriage Cost information; OfEMU = Offline Exposure to Marriage Utility information; OfEMC =

Offline Exposure to Marriage Cost information; RUIE = Relative Utility Information Exposure; RCIE = Relative Cost Information Exposure; “A-B” = B type of A, e.g., “MUV-Em” = Marriage Emotional Utility Value; S = Security; Ec = Economic; FC = Family Continuity; Ps = Psychological; O = Opportunity; Py = Physiological.
