# Supplementary material for: Online exposure to marriage information and marriage expectations of Generation Z in China: The roles of marriage value and relative information exposure
Source: PLoS One. 2025 Oct 27;20(10):e0334596. doi: 10.1371/journal.pone.0334596 (PMC12558505; doi:10.1371/journal.pone.0334596)
Supplement: S3 Table — Notes: The value in parentheses is Exp(B). N = 1390. *p < .05, **p < .01, ***p < .001. MI = Marital Intention; MUV = Marriage Utility Value; MCV = Marriage Cost Value; OEMU = Online Exposure to Marriage Utility information; OEMC = Online Exposure to Marriage Cost information; “A-B” = B type of A, e.g., “MUV-Em” = Marriage Emotional Utility Value; S = Security; Ec = Economic; FC = Family Continuity; Ps = Psychological; O = Opportunity; Py = Physiological. (PDF) [file pone.0334596.s005.pdf]

S3 Table. The regression results of online exposure to marriage information, marriage value and marriage intention

Independent Variable	Logistic	OLS	Logistic	Logistic	OLS	Logistic
	Model1: MI	Model2: MUV	Model3: MI	Model4: MI	Model5: MCV	Model6: MI
Control Variable	YES	YES	YES	YES	YES	YES
OEMU	0.278 (1.320) ***	0.119 ***	0.099 (1.104)			
OEMC				−0.201 (0.818) ***	0.174 ***	0.157 (1.170)
MUV			1.878 (6.541) ***			
MCV						−1.890 (0.151) ***
Nagelkerke R^2/R^2	0.079	0.148	0.318	0.072	0.131	0.265
Independent Variable	Logistic	OLS	Logistic	Logistic	OLS	Logistic
	Model7: MI	Model8: MUV-Em	Model9: MI	Model10: MI	Model11: MUV-S	Model12: MI
Control Variable	YES	YES	YES	YES	YES	YES
OEMU-Em	0.124 (1.132)	0.056 ***	0.053 (1.055)			
OEMU-S				0.163 (1.177) *	0.072 ***	0.120 (1.128)
MUV-Em			1.436 (4.204) ***			
MUV-S						1.202(3.328) ***
Nagelkerke R^2/R^2	0.076	0.096	0.305	0.079	0.153	0.237
Independent Variable	Logistic	OLS	Logistic	Logistic	OLS	Logistic
	Mode13: MI	Model14: MUV-Ec	Model15: MI	Model16: MI	Model17: MUV-FC	Model18: MI
Control Variable	YES	YES	YES	YES	YES	YES
OEMU-Ec	0.244 (1.276) ***	0.099***	0.159 (1.172) *			
OEMU-FC				0.192 (1.211) **	0.134***	0.064 (1.066)
MUV-Ec			1.025 (2.788) ***			
MUV-FC						0.936 (2.549) ***
Nagelkerke R^2/R^2	0.088	0.062	0.188	0.082	0.121	0.211

Independent Variable	Logistic	OLS	Logistic	Logistic	OLS	Logistic
	Model19:MI	Model20: MCV-Ps	Model21: MI	Model22: MI	Model23: MCV-O	Model24: MI
Control Variable	YES	YES	YES	YES	YES	YES
OEMC-Ps	−0.095 (0.909)	0.103 ***	0.132 (1.141)			
OEMC-O				−0.152 (0.859) *	0.123 ***	0.004 (1.004)
MCV-Ps			−1.669 (0.188) ***			
MCV-O						−0.915 (0.400) ***
Nagelkerke R^2/R^2	0.076	0.082	0.277	0.078	0.093	0.179
Independent Variable	Logistic	OLS	Logistic	Logistic	OLS	Logistic
	Model25: MI	Model26: MCV-Ec	Model27: MI	Model28: MI	Model29: MCV-Py	Model30: MI
Control Variable	YES	YES	YES	YES	YES	YES
OEMC-Ec	−0.026 (0.974)	0.156 ***	0.060 (1.062)			
OEMC-Py				−0.277 (0.758) ***	0.228 ***	0.022 (1.022)
MCV-Ec			−0.582 (0.559) ***			
MCV-Py						−1.562 (0.210) ***
Nagelkerke R^2/R^2	0.072	0.055	0.108	0.093	0.215	0.261

Notes: The value in parentheses is Exp(B). N=1390. *p<.05, **p<.01, ***p<.001. MI = Marital Intention; MUV = Marriage Utility Value; MCV = Marriage Cost Value; OEMU = Online Exposure to Marriage Utility information; OEMC = Online Exposure to Marriage Cost information; "A-B" = B type of A, e.g., "MUV-Em" = Marriage Emotional Utility Value; S = Security; Ec = Economic; FC = Family Continuity; Ps = Psychological; O = Opportunity; Py = Physiological.
