# Supplementary material for: Online exposure to marriage information and marriage expectations of Generation Z in China: The roles of marriage value and relative information exposure
Source: PLoS One. 2025 Oct 27;20(10):e0334596. doi: 10.1371/journal.pone.0334596 (PMC12558505; doi:10.1371/journal.pone.0334596)
Supplement: S4 Table — Notes: N = 1261. *p < .05, **p < .01, ***p < .001. EMA = Expected Marriage Age; MUV = Marriage Utility Value; MCV = Marriage Cost Value; OEMU = Online Exposure to Marriage Utility information; OEMC = Online Exposure to Marriage Cost information; “A-B” = B type of A, e.g., “MUV-Em” = Marriage Emotional Utility Value; S = Security; Ec = Economic; FC = Family Continuity; Ps = Psychological; O = Opportunity; Py = Physiological. (PDF) [file pone.0334596.s006.pdf]

S4 Table. The regression results of online exposure to marriage information, marriage value and expected marriage age

	Model1: EMA	Model2: MUV	Model3: EMA	Model4: EMA	Model5: MCV	Model6: EMA
Control Variable	YES	YES	YES	YES	YES	YES
OEMU	−0.026	0.095***	−0.004			
OEMC				0.022	0.161***	−0.004
MUV			−0.231***			
MCV						0.160***
R^2	0.015	0.139	0.062	0.014	0.117	0.050
	Model7: EMA	Model8: MUV-Em	Model9: EMA	Model10: EMA	Model11: MUV-S	Model12: EMA
Control Variable	YES	YES	YES	YES	YES	YES
OEMU-Em	−0.002	0.036*	0.005			
OEMU-S				-0.001	0.057***	0.009
MUV-Em			−0.183***			
MUV-S						−0.164***
R^2	0.013	0.089	0.053	0.013	0.140	0.054
	Mode13: EMA	Model14: MUV-Ec	Model15: EMA	Model16: EMA	Model17: MUV-FC	Model18: EMA
Control Variable	YES	YES	YES	YES	YES	YES
OEMU-Ec	−0.040**	0.091***	−0.030*			
OEMU-FC				−0.027*	0.116***	−0.012
MUV-Ec			−0.105***			
MUV-FC						−0.133***
R^2	0.020	0.064	0.033	0.016	0.117	0.045
	Model19:EMA	Model20: MCV-Ps	Model21: EMA	Model22: EMA	Model23: MCV-O	Model24: EMA
Control Variable	YES	YES	YES	YES	YES	YES
OEMC-Ps	0.012	0.092***	0.001			

OEMC-O				0.015	0.103***	0.000
MCV-Ps			0.123***			
MCV-O						0.143***
R^2	0.014	0.071	0.042	0.014	0.076	0.056
	Model25: EMA	Model26: MCV-Ec	Model27: EMA	Model28: EMA	Model29: MCV-Py	Model30: EMA
Control Variable	YES	YES	YES	YES	YES	YES
OEMC-Ec	0.010	0.158***	0.002			
OEMC-Py				0.019	0.218***	−0.006
MCV-Ec			0.052***			
MCV-Py						0.116***
R^2	0.013	0.060	0.020	0.014	0.195	0.043

Notes: N=1261. **p*<.05, ***p*<.01, ****p*<.001. EMA = Expected Marriage Age; MUV = Marriage Utility Value; MCV = Marriage Cost Value; OEMU = Online Exposure to Marriage Utility information; OEMC = Online Exposure to Marriage Cost information; "A-B" = B type of A, e.g., "MUV-Em" = Marriage Emotional Utility Value; S = Security; Ec = Economic; FC = Family Continuity; Ps = Psychological; O = Opportunity; Py = Physiological.
