# Supplementary material for: Online exposure to marriage information and marriage expectations of Generation Z in China: The roles of marriage value and relative information exposure
Source: PLoS One. 2025 Oct 27;20(10):e0334596. doi: 10.1371/journal.pone.0334596 (PMC12558505; doi:10.1371/journal.pone.0334596)
Supplement: S2 File — (DOCX) [file pone.0334596.s008.docx]

# Scales of This Study

**Marriage Value Scale**

Please rate the extent to which the following statements fit your actual situation. The applicability rating scale is:

1= strongly disagree

2= disagree

3= not sure

4= agree

5= strongly agree

***Marriage Utility Value Scale***

**Marriage Emotional Utility Value.**

Marriage can alleviate loneliness and bring a sense of fulfillment to life.

Marriage can satisfy personal family attachment and give people a sense of belonging.

Marriage can achieve social identity and conform to the traditional values of society and family that individuals should get married at a certain age.

**Marriage Security Utility Value.**

Marriage can reduce the risk of loneliness and aging.

Marriage can avoid the risk of facing difficulties alone.

Marriage can reduce the risk of lovers breaking up.

**Marriage Economic Utility Value.**

Marriage can increase family income.

Marriage can increase household labor.

Marriage can increase an individual's social network and expand their circle.

**Marriage Family Continuity Value.**

Marriage can achieve family prosperity.

Having children after marriage can achieve the inheritance of life.

***Marriage Cost Value Scale***

**Marriage Psychological Cost Value.**

Communication and interaction with my spouse and their relatives after marriage can make me feel anxious.

After marriage, the amount of time spent on myself will be reduced, and personal interests, hobbies, habits, and other freedoms will be restricted.

Getting married is not in line with the current social values of unmarried and infertile young people.

**Marriage Opportunity Cost Value.**

Getting married means giving up on the possibility of finding a better partner in the future.

Marriage will affect my (or my spouse's) career development.

Getting married will affect my (or my spouse's) academic progress.

**Marriage Economic Cost Value.**

Getting married means higher costs such as dowries and purchasing a house.

Getting married means higher daily household expenses.

Getting married means higher elderly care costs of both parents.

**Marriage** **Physiological Cost Value.**

Daily fatigue after marriage can lead to a decrease in physical health.

Having children after marriage can cause harm to women's bodies.

There may be physical or mental domestic violence after marriage.

**Online Exposure to Marriage Information Scale**

Please report the frequency you get the information about some theme through Internet. The applicability rating scale is:

1=never

2=once or twice a year

3=once or twice half a year

4=once or twice a month

5=once or twice a week

6= every day

I get the information of marriage emotional utility (such as the sense of life fulfillment, personal belonging and social identity brought by your spouse) through the Internet.

I get the information of marriage security utility (such as reducing the risk of lonely aging, avoiding difficulties alone, and reducing the risk of lovers breaking up) through the Internet.

I get the information of marriage economic utility (such as increasing family income, increasing family labor, and increasing social contacts) through the Internet.

I get the information of marriage family continuity utility (such as family prosperity and life inheritance) through the Internet.

I get the information of marriage psychological cost (such as anxiety about communication with spouses and their relatives, lack of freedom in marriage constraints, and social disapproval) through the Internet.

I get the information of marriage opportunity cost (such as worries about the choice of a suitable partner, marriage's worries about its own or spouse's career development, and marriage's worries about its own or spouse's learning and improvement) through the Internet.

I get the information of marriage economic cost (such as marriage costs such as bride price, household daily expenses, and the elderly support costs of both parents) through the Internet.

I get the information of marriage physiological cost (such as physical health caused by daily tiredness after marriage, physical harm to women caused by childbirth after marriage, physical or mental domestic violence) through the Internet.

**Offline Exposure to Marriage Information Scale**

I communicate with people around me (such as family, friends, teachers, etc.) about marriage emotional utility (such as the sense of life fulfillment, personal belonging and social identity brought by your spouse) offline.

I communicate with people around me (such as family, friends, teachers, etc.) about marriage security utility (such as reducing the risk of lonely aging, avoiding difficulties alone, and reducing the risk of lovers breaking up) offline.

I communicate with people around me (such as family, friends, teachers, etc.) about marriage economic utility (such as increasing family income, increasing family labor, and increasing social contacts) offline.

I communicate with people around me (such as family, friends, teachers, etc.) about marriage family continuity utility (such as family prosperity and life inheritance) offline.

I communicate with people around me (such as family, friends, teachers, etc.) about marriage psychological cost (such as anxiety about communication with spouses and their relatives, lack of freedom in marriage constraints, and social disapproval) offline.

I communicate with people around me (such as family, friends, teachers, etc.) about marriage opportunity cost (such as worries about the choice of a suitable partner, marriage's worries about its own or spouse's career development, and marriage's worries about its own or spouse's learning and improvement) offline.

I communicate with people around me (such as family, friends, teachers, etc.) about marriage economic cost (such as marriage costs such as bride price, household daily expenses, and the elderly support costs of both parents) offline.

I communicate with people around me (such as family, friends, teachers, etc.) about marriage physiological cost (such as physical health caused by daily tiredness after marriage, physical harm to women caused by childbirth after marriage, physical or mental domestic violence) offline.

**Marriage Expectations**

What age do you plan to get married at?

1=before 24 years old

2=25-29 years old

3=30-34 years old

4=35-39 years old

5=40-44 years old

6=45-50 years old

7=after 50 years old

8=never

* When participants selected “no marriage”, we coded marriage intention as 0; otherwise, it was coded as 1. We then assigned values from 1 to 7 to the expected marriage age ranging from “before 24 years old” to “after 50 years old” in sequential order.
